# Supplementary material for: G-Quadruplex Regulation of VEGFA mRNA Translation by RBM4
Source: Int J Mol Sci. 2022 Jan 11;23(2):743. doi: 10.3390/ijms23020743 (PMC8776124; doi:10.3390/ijms23020743)
Supplement: Supplementary file 1 [file ijms-23-00743-s001.zip › ijms-1534957-supplementary.pdf]

SUPPLEMENTARY DATA

G-quadruplex regulation of mRNA translation of *VEGFA* by RBM4

Kangkang Niu[1,#], Xiaojuan Zhang[1,#], Qisheng Song[2], Qili Feng[1,*]

1 Guangdong Provincial Key Laboratory of Insect Developmental Biology and Applied Technology, Guangzhou Key Laboratory of Insect Development Regulation and Application Research, Institute of Insect Science and Technology, School of Life Sciences, South China Normal University, Guangzhou 510631, China; kkniu@m.scnu.edu.cn (K.N.); 348567235@qq.com (X.Z.)

2 Division of Plant Sciences, University of Missouri, Columbia, MO 65211, USA; songq@missouri.edu (Q.S.)

* Correspondence: qlfeng@scnu.edu.cn.

# There authors contributed equally to this work.

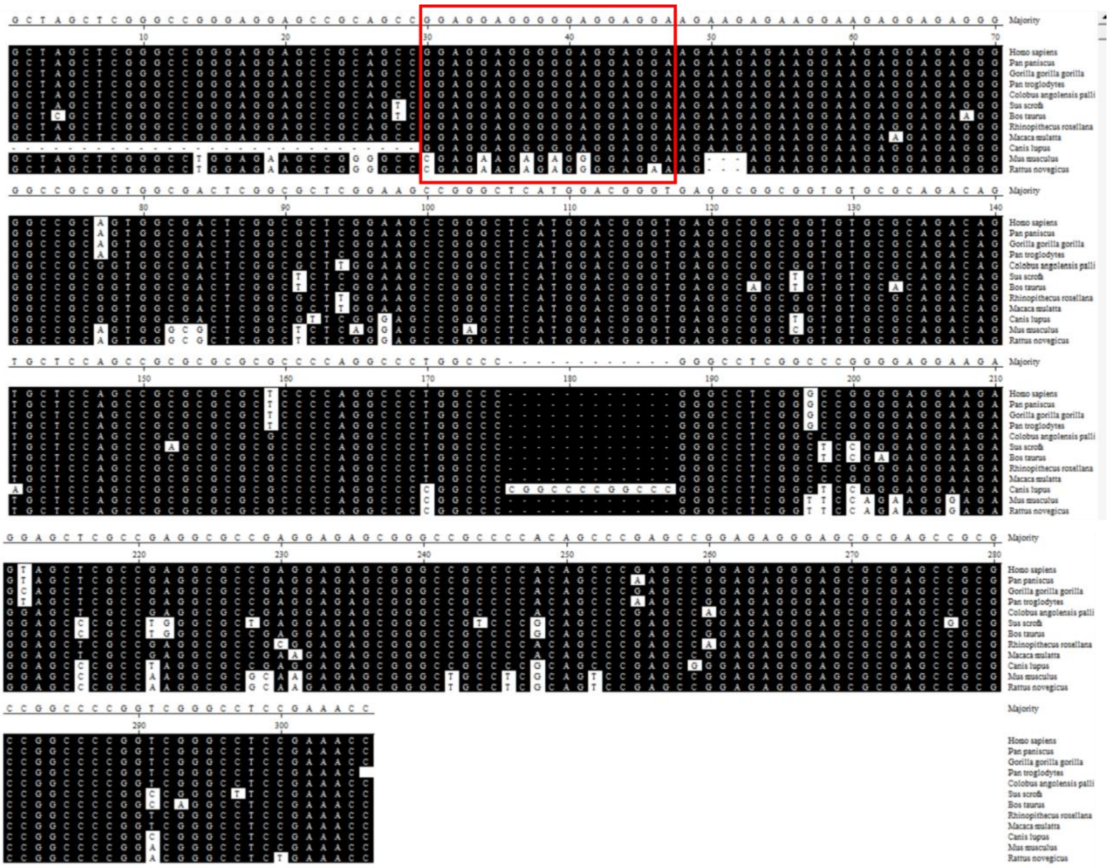

Figure S1. Alignment of sequences of *VEGFA* 5'UTR in different species. The G4 sequences were boxed in red.
